# Supplementary figures and images for: A systemic review and network meta-analysis of accuracy of intraocular lens power calculation formulas in primary angle-closure conditions
Source: PLoS One. 2022 Oct 14;17(10):e0276286. doi: 10.1371/journal.pone.0276286 (PMC9565378; doi:10.1371/journal.pone.0276286)

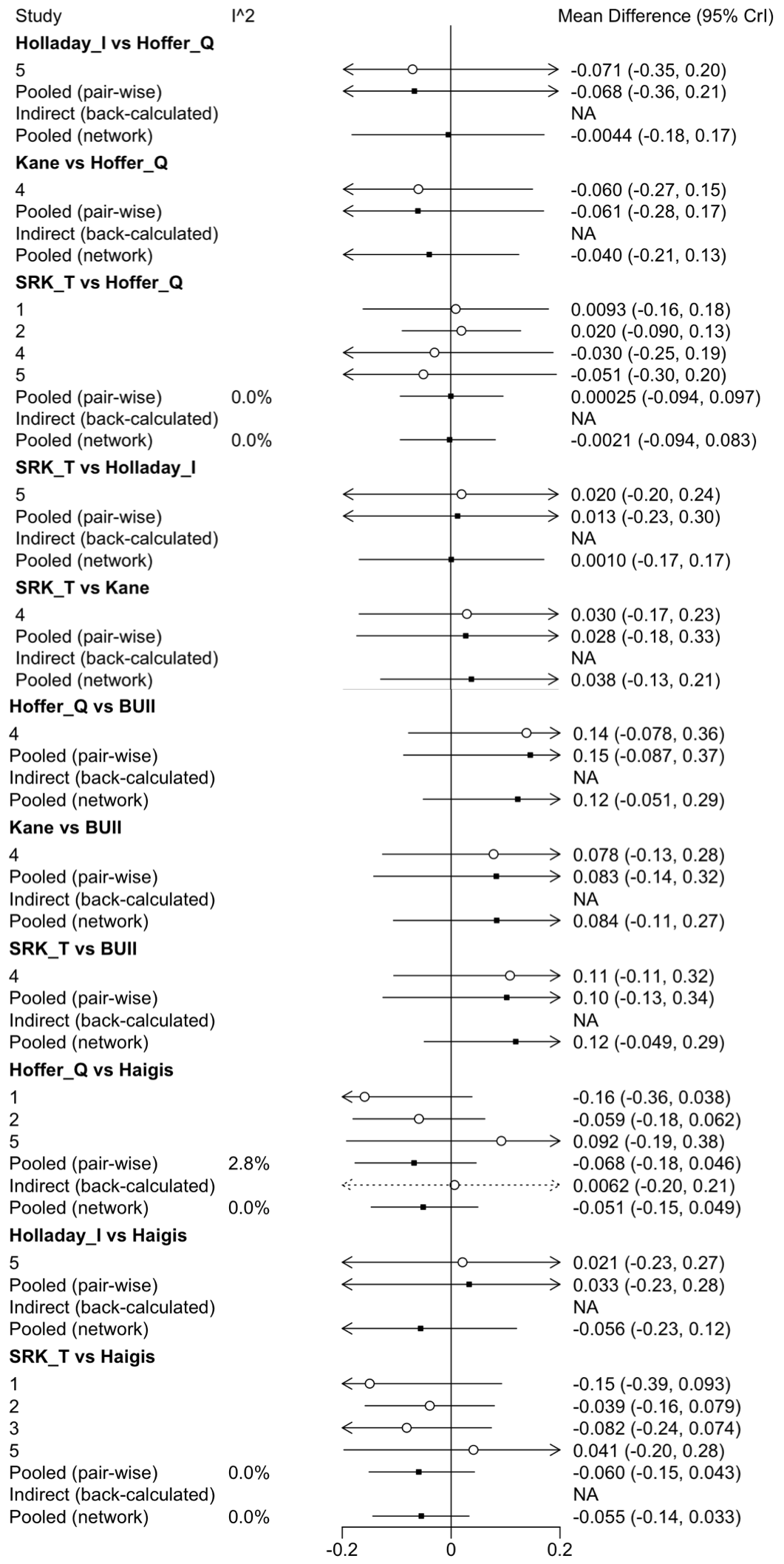

Supplement: S1 Fig — Between-study heterogeneity of MAE was measured using mtc.anohe package in R, with the results showing consistency in both pair-wise and network comparison. (TIFF) [file pone.0276286.s002.tiff]

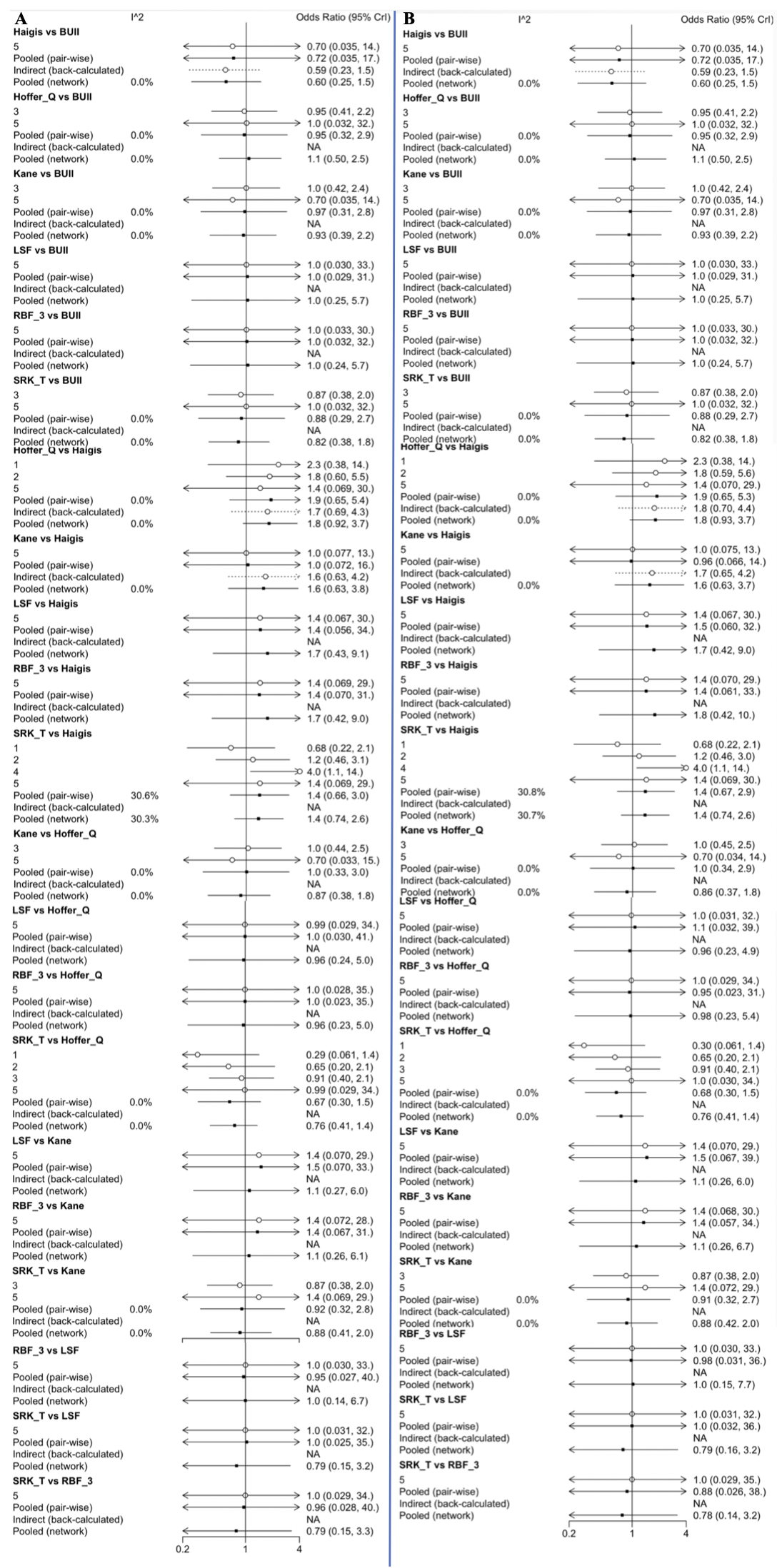

Supplement: S2 Fig — Between-study heterogeneity of A. % ±0.50 D and B. % ±1.00 D was measured using mtc.anohe package in R, with the results showing consistency in all the network comparison. (TIFF) [file pone.0276286.s003.tiff]

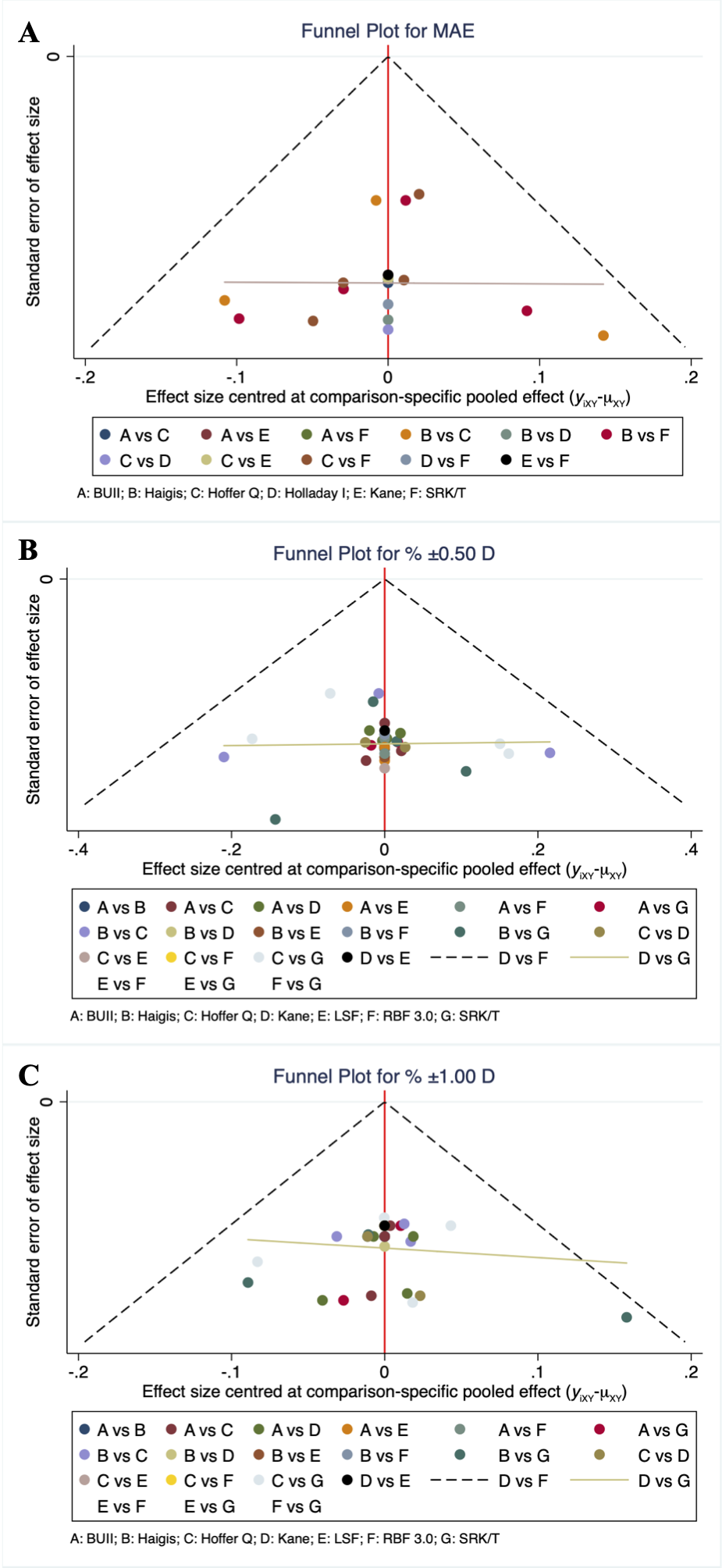

Supplement: S3 Fig — Risk of publication bias of A. MAE, B. % ±0.50 D and C. % ±1.00 D was measured using network funnel plot showing symmetry, indicating relatively small publication bias. (TIFF) [file pone.0276286.s004.tiff]

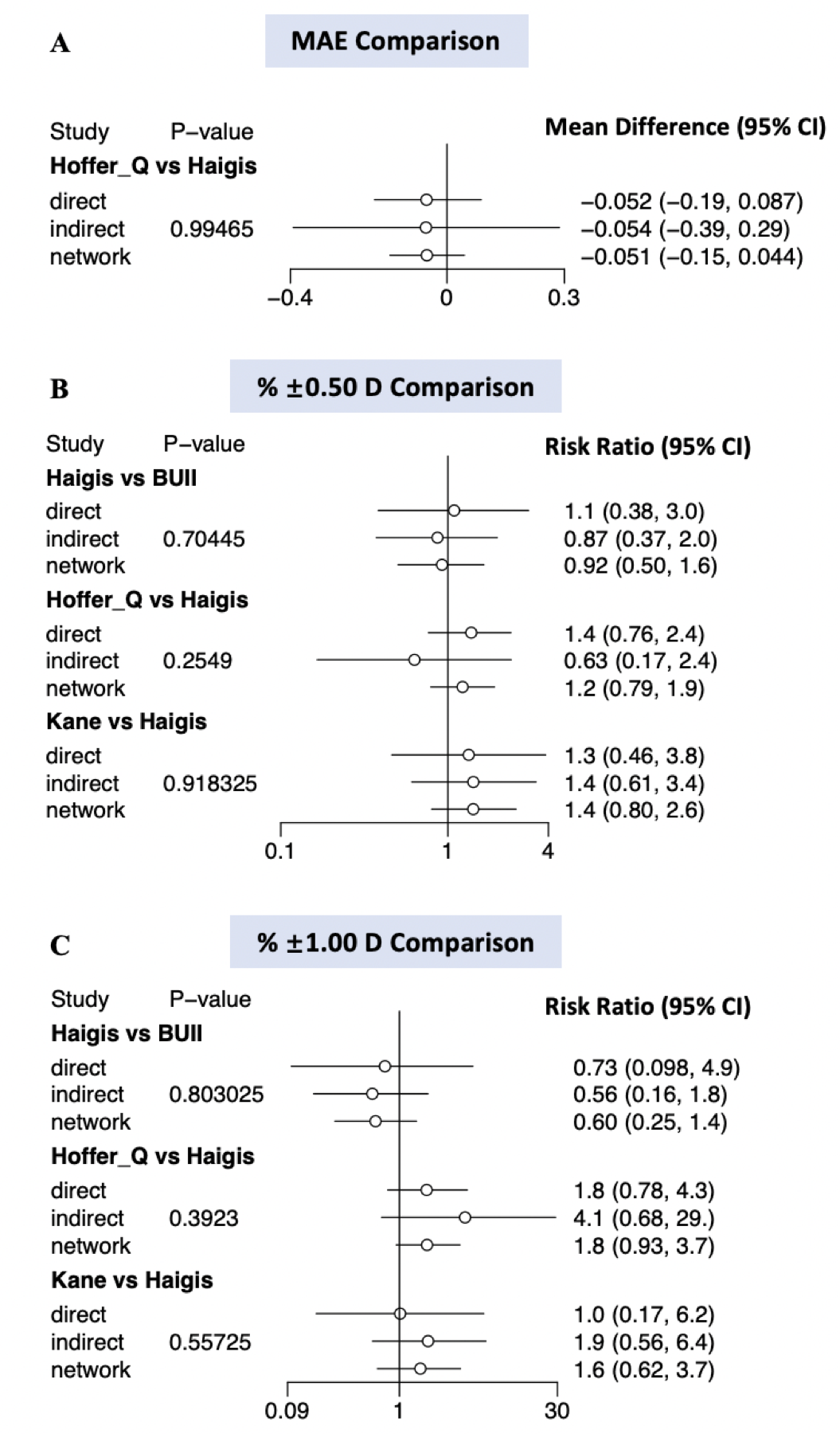

Supplement: S4 Fig — The decision rule selected Haigis-Hoffer Q comparisons for MAE (A), Haigis-BUII, Haigis-Hoffer Q and Haigis-Kane comparisons for % ±0.50 D (B) and % ±1.00 D (C) because of presence of both direct and indirect comparisons in these pairs. As reflected by P-value, the direct and indirect comparisons were in agreement of each other. MAE: mean absolute error; BUII: Barrett Universal II. (TIFF) [file pone.0276286.s005.tiff]
