# Supplementary material for: A systemic review and network meta-analysis of accuracy of intraocular lens power calculation formulas in primary angle-closure conditions
Source: PLoS One. 2022 Oct 14;17(10):e0276286. doi: 10.1371/journal.pone.0276286 (PMC9565378; doi:10.1371/journal.pone.0276286)
Supplement: S1 Table — (DOCX) [file pone.0276286.s006.docx]

**Supplementary Table 1A Details of Search Strategy Source: Pubmed; Searched on: 30, Aug, 2022;**

| Search | Query | Items |
| --- | --- | --- |
| [#1](https://www.ncbi.nlm.nih.gov/pubmed/advanced) | “glaucoma, angle-closure”[Mesh] | 3824 |
| #2 | “angle closure”[Title/Abstract] | 4712 |
| #3 | “angle-closure”[Title/Abstract] | 4712 |
| #4 | [#1](https://www.ncbi.nlm.nih.gov/pubmed/advanced) OR [#2](https://www.ncbi.nlm.nih.gov/pubmed/advanced) OR #3 | 5878 |
| #5 | “cataract” [Mesh] | 31659 |
| #6 | “cataract” [Title/Abstract] | 55267 |
| #7 | “lenses, intraocular”[Mesh] | 16805 |
| #8 | “IOL”[Title/Abstract] | 11517 |
| #9 | “intraocular lens”[Title/Abstract] | 15718 |
| #10 | #5 OR #6 OR #7 OR #8 OR #9 | 77609 |
| #11 | #4 AND #10 | 1064 |

**Supplementary Table 1B Details of Search Strategy Source: Web of Science; Searched on: 30, Aug, 2022;**

| **Search** | **Query** | **Items** |
| --- | --- | --- |
| [#1](https://www.ncbi.nlm.nih.gov/pubmed/advanced) | TI=(angle closure) OR AB=(angle closure) | 17771 |
| #2 | TI=(angle-closure) OR AB=(angle-closure) | 6469 |
| #3 | [#1](https://www.ncbi.nlm.nih.gov/pubmed/advanced) OR [#2](https://www.ncbi.nlm.nih.gov/pubmed/advanced) | 17771 |
| #4 | TI=(cataract) OR AB=(cataract) | 94473 |
| #5 | TI=(IOL) OR AB=(IOL) | 17166 |
| #6 | TI=(intraocular lens) OR AB=(intraocular lens) | 38540 |
| #7 | #4 OR #5 OR #6 | 119770 |
| #8 | #3 AND #7 | 1465 |

**Supplementary Table 1C Details of Search Strategy Source: Cochrane Library; Searched on: 30, Aug, 2022**

| **Search** | **Query** | **Items** |
| --- | --- | --- |
| [#1](https://www.ncbi.nlm.nih.gov/pubmed/advanced) | (angle closure):ti,ab,kw | 674 |
| #2 | (angle-closure):ti,ab,kw | 532 |
| #3 | #1 OR #2 | 674 |
| #4 | (cataract):ti,ab,kw | 8419 |
| #5 | (IOL):ti,ab,kw | 2339 |
| #6 | (intraocular lens):ti,ab,kw | 3695 |
| #7 | #4 OR #5 OR #6 | 9682 |
| #8 | #3 AND #7 | 203 |
