# Supplementary material for: A systemic review and network meta-analysis of accuracy of intraocular lens power calculation formulas in primary angle-closure conditions
Source: PLoS One. 2022 Oct 14;17(10):e0276286. doi: 10.1371/journal.pone.0276286 (PMC9565378; doi:10.1371/journal.pone.0276286)
Supplement: S2 Table — Level of evidence as recommended by the Oxford Centre for Evidence-based Medicine. *Retrospective Observational Studies. (PDF) [file pone.0276286.s007.pdf]

Study	Randomization	Blinding	Free of selective reporting	Other potential bias	Level of evidence
**Joo et al., 2011**	None*	No	Yes	No	IIb
**Seo et al., 2016**	None*	No	Yes	No	IIb
**Song et al., 2018**	None*	No	Yes	No	IIb
**Li et al., 2021**	None*	No	Yes	No	IIb
**Lee et al., 2021**	None*	No	Yes	No	IIb
**Hou et al., 2021**	None*	No	Yes	No	IIb

Level of evidence as recommended by the Oxford Centre for Evidence-based Medicine.
*Retrospective Observational Studies.
